# Experimental parasite infection reveals costs and benefits of paternal effects

**DOI:** 10.1111/ele.12344

**Published:** 2014-08-28

**Authors:** Joshka Kaufmann, Tobias L Lenz, Manfred Milinski, Christophe Eizaguirre

**Affiliations:** 1 Department of Evolutionary Ecology, Max Planck Institute for Evolutionary BiologyPlön, 24306, Germany; 2 Division of Genetics, Brigham and Women's Hospital, Harvard Medical SchoolBoston, MA, 02115, USA; 3 GEOMAR, Helmholtz Centre for Ocean ResearchKiel, 24105, Germany; 4 School of Biological and Chemical Sciences, Queen Mary University of LondonLondon, E1 4NS, UK

**Keywords:** Host–parasite interaction, *in vitro* fertilisation, paternal effects, sperm phenotype, three-spined stickleback

## Abstract

Forces shaping an individual's phenotype are complex and include transgenerational effects. Despite low investment into reproduction, a father's environment and phenotype can shape its offspring's phenotype. Whether and when such paternal effects are adaptive, however, remains elusive. Using three-spined sticklebacks in controlled infection experiments, we show that sperm deficiencies in exposed males compared to their unexposed brothers functionally translated into reduced reproductive success in sperm competition trials. In non-competitive fertilisations, offspring of exposed males suffered significant costs of reduced hatching success and survival but they reached a higher body condition than their counterparts from unexposed fathers after experimental infection. Interestingly, those benefits of paternal infection did not result from increased resistance but from increased tolerance to the parasite. Altogether, these results demonstrate that parasite resistance and tolerance are shaped by processes involving both genetic and non-genetic inheritance and suggest a context-dependent adaptive value of paternal effects.

## Introduction

Understanding non-Mendelian modes of inheritance, such as parental effects, has become an important theme in evolutionary biology (Bonduriansky 2012; Rando 2012). Parental effects are defined as the influence of parental phenotypes on their offspring's phenotype beyond the direct effects of genetic inheritance (Mousseau *et al.* 2009; Wolf & Wade 2009). While increasingly acknowledged as an important factor, there is still controversy over the general adaptive value of parental effects (Marshall & Uller 2007). To be selected for, parental effects have to be, on average, at least slightly beneficial, however, on a short time scale, they can be beneficial to the parents, the offspring, both or neither of them (Marshall & Uller 2007). The adaptive value of a parental effect is expected to depend on the distribution of costs and benefits across parental and offspring generations and more importantly depends on the ecological context (Mousseau & Fox 1998; Marshall 2008). Adaptive parental effects are expected to evolve when the selective pressures are both variable and predictable (Burgess & Marshall 2014). Despite significant progress, the context dependence nature of adaptive parental effects is still poorly understood. Furthermore, even though studies have mainly focused on maternal effects, there is growing evidence for variation in offspring phenotypes that may be attributed specifically to paternal effects (Mousseau & Fox 1998; Curley *et al.* 2011; Rando 2012). Studying paternal effects practically facilitates the experimental testing of adaptive non-genetic transmission, because, in contrast to the mother (e.g. through placenta, egg yolk, milk), the physiological links between father and offspring are generally very limited and can be more easily controlled (Curley *et al.* 2011; Rando 2012).

To assess the adaptive value and context dependence of a paternal effect experimentally, it is necessary to manipulate exactly the same selective pressure in both parental and offspring generations. To this end, experimental exposure to parasites is ideal, given: (1) their ubiquitous presence in nature (Moore 2002) (2) their known fluctuating dynamics (Decaestecker *et al.* 2007) and (3) their detrimental effects on host condition and reproductive success (Kalbe *et al.* 2009; Schulenburg & Kurtz 2009). Genes responding to parasite-mediated selection increase immunological resistance against the parasite and reduce the likelihood of infection (Sorci *et al.* 1997; Eizaguirre & Lenz 2010; Eizaguirre *et al.* 2012). On the other hand, selection can also lead to increased tolerance of infection (Råberg *et al.* 2007; Sorci 2013). While some recent studies have shown that transgenerational immune priming can affect survival, growth and immune responses during parasite or immune challenge (Gallizzi *et al.* 2008; Linder & Promislow 2009; Sadd & Schmid-hempel 2009; Roth *et al.* 2010, 2012), our understanding of the context dependence of adaptive paternal effects and their consequences on resistance, tolerance and more broadly on host–parasite interactions are still poorly understood.

The three-spined stickleback (*Gasterosteus aculeatus* L.) is an established model species for studying the genetic basis of parasite resistance (Wegner *et al.* 2003; Barber 2013) and its Mendelian inheritance (e.g. Eizaguirre *et al.* 2012). Here, we used this model species to investigate whether paternal effects can be expressed under experimental parasite pressure. Specifically, we estimated the effect of parasite exposure across two generations of three-spined sticklebacks, exposed to a standardised dose of a common stickleback parasite, the nematode *Camallanus lacustris*. We produced maternal half-sibships, each sired by one exposed and one unexposed male. The two sires of each half-sibship pair were brothers to reduce the well-documented variation due to classical genetic inheritance. We then studied how paternal infection affected early life-history traits and parasite resistance in the offspring generation. As males mainly contribute semen to the next generation, sperm represents the best candidate for functionally mediating paternal effects (Crean *et al.* 2012; Rando 2012; Bromfield *et al.* 2014). For this, we estimated variability of sperm traits under parasite infection and their consequences in competitive and non-competitive *in vitro* fertilisation experiments.

## Materials and methods

### Parasite exposure of laboratory-bred fathers

We dissected larvae of the nematode *Camallanus lacustris* from gravid female parasites collected from intestines of adult perches *Perca fluviatilis* from the vicinity of the stickleback population (Dieksee, 54°9′32.82″, 10°29′47.63″, Germany). This parasite is highly prevalent in the stickleback fish population (Kalbe *et al.* 2002; Eizaguirre *et al.* 2011), negatively affects their growth (Eizaguirre *et al.* 2012), and is known to activate their immune system (Krobbach *et al.* 2007) as well as to select for resistance alleles at major histocompatibility complex genes (Eizaguirre *et al.* 2012). As this parasite is trophically transmitted, we used copepods (*Macrocyclops albidus*) from a parasite-free laboratory culture as intermediate hosts (van der Ven *et al.* 2000). We exposed groups of 100 copepods to 400 and 500 *C. lacustris* larvae for the paternal exposure and the offspring exposure respectively. The number of larvae in the body cavity of each copepod was counted under a microscope to standardise the number of parasites each fish was exposed to. This manipulation guaranteed that the observed infection was directly linked to the immunocompetence of the fish and not confounded by the number of parasites the fish were exposed to (Eizaguirre *et al.* 2012).

We bred 10 full-sib families of three-spined sticklebacks, subsequently referred to as the G1 generation, by randomly pairing males and females from a natural lake population (Grosser Plöner See, 54°9′21.16″ N, 10°25′50.14″ E, Germany). The fish from those families were kept under controlled laboratory conditions and were parasite free at the beginning of the experiment. Male juveniles of each G1 family were randomly assigned to one of two treatments: parasite exposure or control (i.e. no exposure). We exposed males from the ‘exposure’ treatment group twice to exactly six *C. lacustris* larvae (in copepods), whereas control males only received uninfected copepods. All G1 fish were transferred through artificial fall, winter and spring conditions in the laboratory to induce sexual maturation. Sixteen weeks after exposure, the G1 males (exposed and unexposed) were separated in single 16-L aquaria with nesting material, whereas the G1 females were maintained in group aquaria (Sommerfeld *et al.* 2008). All individuals were fed *ad libitum* with frozen and live chironomid larvae. Males were inspected daily and nest quality of all males was evaluated following Jäger *et al.* (2007). Only pairs (i.e. brothers) of reproductively active males (courting behaviour, each maintaining a nest of high quality for at least 2 days) were used in the experiments. For each trial, the selected males were sacrificed by a cut in the brain. After sperm collection (see below), the entire intestinal tract of each male was screened for *C. lacustris* under a dissection microscope (Kalbe *et al.* 2002). All exposed males were infected with at least one worm.

The parasite exposure treatment in the G1 generation could potentially result in an unintended and confounding selection bias in male quality between the treatment groups. This is because parasite exposure is known to affect mortality and reproductive behaviour. To control for this unintended bias, we tested whether more exposed than unexposed G1 males were excluded during the course of the experiment, for instance, due to low nest quality. However, we did not find significant differences between exposed and unexposed G1 males in mortality, nest building behaviour, or the manifestation of courtship behaviour (all *P* > 0.49; see Table S1).

### Sperm isolation and measurements

For both types of *in vitro* fertilisation experiments, testes of G1 males were freshly dissected, weighed and transferred to a 40 μm microcell strainer sieve with 300 μL Hank's balanced salt solution (HBSS) solution (HBSS, Sigma-Aldrich, Munich, Germany). Sperm suspension was prepared by gently mashing each testes through a cell strainer using a plastic stamp and rinsing the sieve twice with 300 μL HBSS solution. Three microlitres of the resulting suspension was transferred to a counting chamber (standard count four-chamber slide, 20 μm depth, Leja, Nieuw Vennep, Netherlands) under an Olympus CX41 microscope at 100× magnification. To quantify spermatozoa concentration and velocity, we used computer-assisted sperm analysis using a Hamilton-Thorne CEROS camera setup and the Animal Mobility software (Hamilton Thorne Biosciences, Beverly, MA, USA). We recorded the total number of sperm, motile sperm number and the following sperm motion parameters: Beat-cross frequency as well as curvilinear (VCL), straight-line (VSL) and average-path velocity (VAP) (Kime *et al.* 2001). We recorded six measurements of each sperm characteristic per individual (three separate areas from each of two slide chambers) and used the average value in subsequent analyses.

### *In vitro* sperm competition experiments

To test for the consequences of parasite exposure on the functional variation of fertilisation, we prepared 15 sperm competition assays between sperm extracted from one exposed and one unexposed male of the same laboratory-bred G1 family. Using brothers for these experiments reduces the effect of genetic variation on sperm phenotypes, sperm competition outcome and offspring phenotype. For each test, we fertilised the eggs of a random female (taken from the same laboratory-bred G1 generation but not from the males’ family) with 50 μL of sperm solution from each of the two brothers in 5 mL of fresh water. Using the same individuals, we also performed matched sperm competition assays where total sperm concentration was adjusted to the lowest concentration of the two males. Five days after fertilisation, the eggs were sampled for DNA analysis. DNA extraction was performed using the Invisorb® DNA Tissue HTS 96 Kit (Invitek, Berlin, Germany) on a TECAN FreedomEvo robot platform. All eggs were genotyped at 15 microsatellite loci (Kalbe *et al.* 2009) for paternity analysis (*n* = 1157, mean of eggs per test 39 ± 14 SD). We performed genotyping using GeneMarker 1.85 (Softgenetics LLC, State College, PA, USA) and individual parentage analysis using CERVUS v3.0.3 (Field Genetics Ltd, Kalinowski *et al.* 2007). The most likely father was determined based on the exclusion probabilities and LOD score ratios between the two putative sires (mean paternal assignment of 93.38%).

### Production of offspring generation

To test for the impact of parasite exposure on fertilisation success and for paternal effects *per se*, we also performed *in vitro* fertilisations in a non-competitive split-clutch design. Each maternal half-sibship pair was mothered by one G1 female and sired by two G1 brothers, one exposed and one unexposed. To control for potential family effects, the parents originated from a total of 10 different G1 laboratory-bred families. In total, we produced 53 maternal half-sibship pairs (range: 2–9; average: 5 per G1 family), subsequently referred to as the G2 generation. For this we randomly selected a G1 gravid female (not the same family as the males’) and two reproductively active brothers. The females’ eggs were stripped carefully into a dry sterile Petri dish (90 × 15 mm). We divided each clutch evenly into two halves: One half was fertilised with 100 μL of sperm solution from the G1 male exposed to the parasite and the other half was fertilised with 100 μL of sperm solution from the unexposed male. Eggs and sperm were left for 20 min at 18°C to assure complete fertilisation. Five days after fertilisation, we counted the number of developing, unfertilised and undeveloped eggs under a laboratory microscope. We characterised unfertilised eggs by the sole presence of lipid droplets and undeveloped eggs by a delayed developmental stage as well as the absence of a heartbeat 5 days post fertilisation (Fig. S1).

### Offspring care and exposure

To estimate juvenile mortality in the G2 generation, we monitored the presence of dead juveniles at least three times a week for 6 months. After this period, we randomly selected 15 maternal half-sib G2 families (representing 5 of the 10 initially produced G1 families) to challenge them with the same nematode parasite as the G1 fathers. For this, we randomly assigned fish of both sexes from each G2 family either to the parasite exposure treatment (9–10 fish per family) or to the control treatment (5–6 fish per family). The total number of fish was 475. Prior to the experimental treatment, fish were measured, weighed and a spine was clipped for later identification. The methods of G2 parasite exposure and fish dissections were strictly the same as in the parental G1 generation except that G2 offspring from the ‘exposure’ treatment were exposed to exactly seven *C. lacustris* larvae each. We then transferred them in groups of 26 fish to 16-L tanks, mixed by paternal treatment, experimental treatment and family to avoid confounding tank effects. We used DNA fingerprinting based on 11 microsatellite loci (Kalbe *et al.* 2009) on spine and fin samples (before and after treatment respectively) to identify G2 individuals with their respective treatments at the end of this double-blind experimental setup. All G1 and G2 fish were laboratory bred and thus parasite free before exposure to *C. lacustris*.

### Statistical analysis

#### Effect of parasite exposure on sperm phenotype and functional competitiveness

All statistical tests were conducted in R v 3.0.3 (R Development Core Team 2014). We tested differences in testes mass and sperm characteristics (velocities and concentration) between exposed and unexposed males using a linear model with treatment and testes mass as fixed effects. We estimated the paternity of the unexposed G1 male in each of the 15 sperm competition trials and tested this value against 50% (representing random fertilisation) using one sample *t*-tests.

#### Cost of paternal exposure on offspring early life-history traits

The proportion of unfertilised and undeveloped eggs was calculated over the total clutch size. Juvenile mortality was calculated based on the total number of dead G2 juveniles over the initial number of developed eggs per clutch. We tested differences in the proportion of unfertilised eggs, undeveloped eggs and juvenile mortality between clutches sired by exposed or unexposed G1 males using non-parametric Wilcoxon signed-rank tests (wilcox.test function in R).

#### Effect of paternal exposure on offspring resistance

Resistance is defined as the ability of hosts to suppress the establishment of parasites and thus limit parasite load (Råberg *et al.* 2009; Sorci 2013). We tested the effects of paternal G1 exposure on the likelihood of infection (infected vs. uninfected, n_exposed_ = 223) and on infection intensity (number of worms in infected individuals) in the G2 fish using generalised linear mixed effect models (glmer function in R). The full model included sex, G2 size before exposure and paternal G1 treatment (exposed vs. control) as fixed effects, and maternal G2 half-sibship identity as random effect to account for non-independence between the two paired maternal half-sibships. Infection probability was fitted with a binomial (log-odds link function) distribution and infection intensity fitted with a Poisson distribution (log link function). The significance of the paternal effect was tested by comparing models with or without the paternal G1 treatment variable using likelihood ratio tests (using anova function in R). We did not find evidence for over-dispersion in our models (Table S2).

#### Effect of paternal exposure on offspring tolerance

Tolerance is defined as the ability of hosts to limit the physiological costs caused by a given parasite burden or, *sensu stricto*, as the reaction norm of host fitness and condition over parasite burden (Råberg *et al.* 2009; Sorci 2013). In our experiment, parasite-related paternal effects could not only be expressed through resistance but also through increased tolerance, where G2 fish sired by exposed G1 males would suffer less from parasite-induced fitness consequences than their counterparts sired by unexposed G1 males. We thus tested whether infection with *C. lacustris* affected body condition in G2 fish differently with respect to the paternal G1 treatment. Body condition of the G2 fish, an estimate of fish health and a predictor of energy reserves and reproductive success, was calculated using the residuals from the regression of body mass on body length (Chellappa *et al.* 1995). The linear mixed effect model (nlme function in R) included G2 body condition as dependent variable, sex, G2 treatment (exposed vs. control), paternal G1 treatment (exposed vs. control) and their interactions as fixed effects as well as maternal G2 half-sibship identity as a random effect.

Fish dissection showed that approximately half of the exposed G2 fish did not harbour any parasite at the end of the experiment. Since the actual moment of infection and the continuing interaction with an established parasite are two inherently different processes, we hypothesised that the cost of parasite infection might be very different between infected and exposed but uninfected G2 individuals. Hence, we tested the effect of paternal G1 exposure on tolerance only in exposed G2 offspring, split for exposed-uninfected and exposed-infected offspring. For this we ran the same linear mixed effect model on G2 body condition as above, but focusing only on exposed G2 fish (exposed-uninfected vs. exposed-infected) instead of all fish.

To investigate in which way paternal G1 exposure affected offspring tolerance, we tested how the relationship between G2 body condition and infection intensity was affected by paternal G1 exposure. This was tested in a linear mixed model on G2 body condition with paternal G1 treatment and the interaction between paternal G1 treatment and G2 infection intensity as fixed effects. Maternal half-sibship identity was set as a random effect.

## Results

### Effect of parasite exposure on sperm phenotype and functional competitiveness

**Figure 1:**
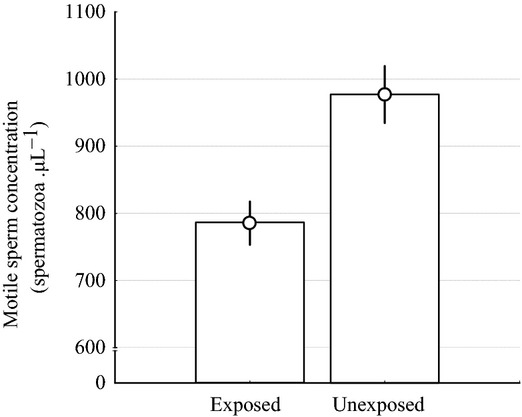
Parasite infection induces sperm deficiency. Concentration of motile spermatozoa per μL in male sticklebacks experimentally infected with the nematode *Camallanus lacustris* and in uninfected (unexposed) males. Error bars represent ± 1 SE.

All exposed G1 males were infected with at least one parasitic worm. We did not find significant differences in testes mass, total sperm concentration or measures of sperm velocity between exposed and unexposed males (testes mass: *P* = 0.13; total sperm concentration: *P* = 0.07; all velocities: *P* > 0.2). However, motile sperm concentration was found to be significantly lower in exposed males than in unexposed males (*F*_1,122_=4.595, *P* = 0.034; Fig. 1). This difference translated into an advantage for the unexposed G1 males, which fertilised on average 65.65% of the eggs. This value was significantly higher than an evenly shared paternity (t_d.f.=14_ = 2.181, *P* = 0.023), but not so when total sperm concentration was experimentally matched between brothers (t_d.f.=13_ = 1.003, *P* = 0.167). These results suggest a reduction in the concentration of motile sperm in response to infection (Pearson's correlation between total and motile sperm concentration: *r* = 0.908, *P* < 0.001).

### Cost of paternal exposure on offspring early life-history traits

**Figure 2:**
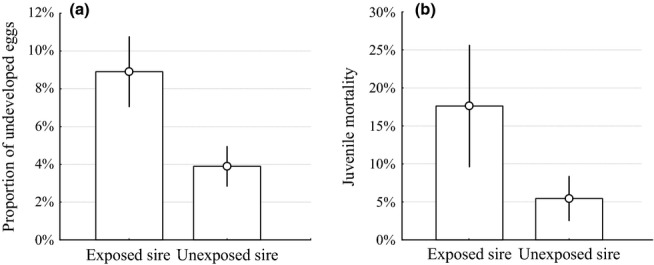
Transgenerational effects of paternal parasite infection on (a) the proportion of undeveloped eggs and (b) the proportion of dead juveniles in maternal half-sibships sired by exposed or unexposed fathers. Error bars represent ± 1 SE.

In non-competitive fertilisation trials, we did not observe significant differences in fertilisation rates between clutches sired by exposed or unexposed males (Wilcoxon signed-rank test: *n* = 53, T = 181, Z = 0.192, *P* = 0.848). However, eggs fertilised by G1 males that were exposed to parasites suffered higher rates of developmental failures than the ones fertilised by unexposed G1 males, resulting in lower hatching success (Wilcoxon signed-rank test: n_clutches_ = 53, n_eggs_ = 4316, T = 157, Z = 2.765, *P* = 0.006; Fig. 2a). Furthermore, larvae of exposed G1 males also showed a higher mortality rate (Wilcoxon signed-rank test: n_clutches_ = 50, n_developed eggs_ = 3602, T = 158.5, Z = 2.912, *P* = 0.004; Fig. 2b). Motile sperm concentration, zygote and juvenile mortality were not significantly correlated among each other (see Table S3).

### Effect of paternal exposure on offspring resistance

We found no significant differences between surviving G2 individuals sired by exposed or unexposed G1 males in their probability to become infected when exposed to the same parasite as the paternal generation (likelihood ratio test (LRT), n_exposed_ = 223, 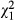 = 3.599, *P* = 0.165, Table S4 and Fig. S2) or in infection intensity (the number of parasites when infected, LRT, n_infected_ = 113, 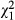 = 0.061, *P* = 0.97, Table S5). Notably, 43% of the variation in the likelihood of being infected was attributable to maternal half-sibship origin.

### Effect of paternal exposure on offspring tolerance

**Figure 3:**
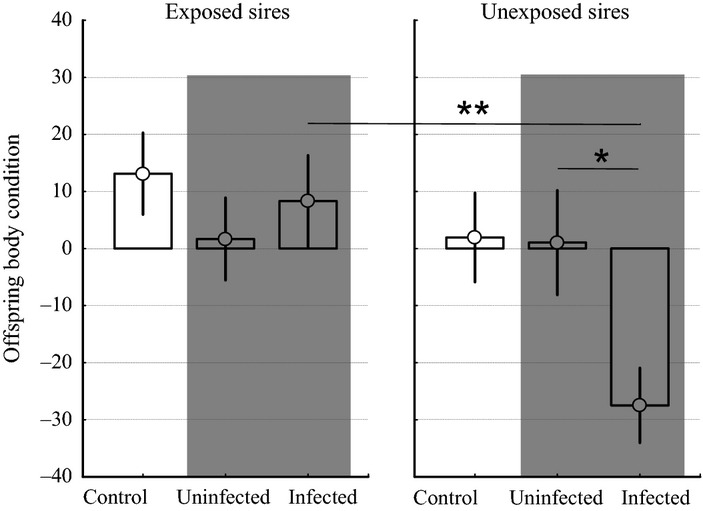
Transgenerational effects of paternal parasite exposure on body condition at the end of the experiment. Body condition is an estimate of fish health and is calculated using the residuals from the regression of body mass on body length. Shown are the means of body condition in control, uninfected (i.e. exposed but non-infected) and infected offspring, sired by either exposed or unexposed fathers. Error bars represent ± 1 SE. The shaded data indicates the comparison of exposed-uninfected and exposed-infected fish. Symbols represent significant differences between experimental groups based on Tukey *post hoc* tests (*P = 0.048; **P = 0.003).

Prior to experimental treatment, offspring sired by unexposed males had higher body condition than offspring sired by exposed males (*F*_1,358_ = 4.32, *P* = 0.038). After the experimental exposure period, we found significant effects of paternal G1 treatment and G2 treatment on G2 body condition. On the one hand, offspring sired by exposed G1 males achieved a higher body condition than their counterparts sired by unexposed G1 males (*F*_1,471_ = 8.74, *P* = 0.003; Table S5 and Fig. S3). On the other hand, experimental parasite exposure significantly reduced body condition in G2 fish (*F*_1,471_ = 6.42, *P* = 0.012; Table S6, Fig. 3). Noteworthy, 38% of the variation in body condition at the end of the experiment was attributable to maternal half-sibship identity.

As exposed G2 fish encompassed both infected and uninfected individuals in approximately equal proportions, we additionally focused on the variation in G2 body condition in response to G1 paternal effects between exposed-infected and exposed-uninfected individuals. Here, we found a significant interaction between paternal G1 treatment and G2 infection status on G2 body condition (*F*_1,282_ = 4.14, *P* = 0.043; Table 1, grey shades in Fig. 3): G2 fish sired by unexposed males suffered significantly from the cost of parasite infection (Tukey *post hoc* test, Z = 2.58, *P* = 0.048), whereas G2 fish sired by exposed males did not (Tukey *post hoc* test, Z = −0.25, *P* = 0.995). This result seemed to be mainly driven by infected G2 fish sired by unexposed G1 males, which showed a significantly lower body condition than their counterparts from exposed G1 males (Tukey *post hoc* test, Z = −3.47, *P* = 0.003, Fig. 3), while paternal G1 exposure did not significantly affect body condition in uninfected individuals (Tukey *post hoc* test, Z = −0.71, *P* = 0.894). This suggests that beneficial effects of paternal exposure are only expressed in offspring upon challenge by the selective parasite.

**Table 1:** Effects of paternal exposure, offspring infection status (infected vs. exposed but uninfected) and sex on individual body condition

Effect	d.f.	*F* value	*P*
Paternal exposure	1, 282	8.161	0.005
Offspring infection	1, 282	2.551	0.111
Offspring sex	1, 282	0.505	0.478
Paternal exp. × Offspring infection	1, 282	4.144	0.043
Maternal half-sibship (random effect)	Variance = 36.44%		

The statistical table shows the outcome of a linear mixed model on individual body condition at the end of the experiment. The variation imputed to the random effect was estimated based on the ratio of the variance due to this effect over the total variance (d.f., degrees of freedom)

**Figure 4:**
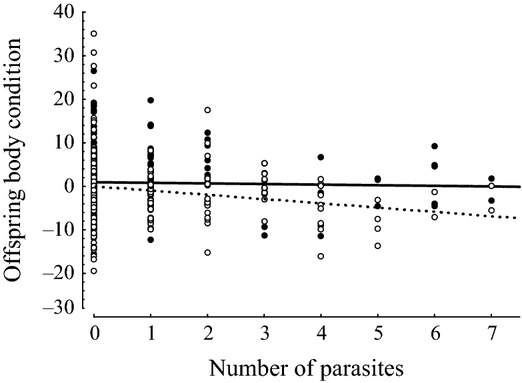
Transgenerational effects of paternal parasite exposure on the relation between offspring body condition and infection intensity (i.e. tolerance). The black circles and the solid linear regression line represent exposed fish sired by exposed fathers and the white circles and the dashed linear regression line represent exposed fish sired by unexposed fathers.

To further dissect this effect, we investigated tolerance as the relationship between offspring body condition and infection intensity, with respect to paternal exposure. We found a significant interaction between paternal G1 exposure treatment and the number of established parasites in G2 fish on body condition (*F*_2,281_ = 4.11, *P* = 0.017, Table 2, Fig. 4): G2 fish sired by unexposed G1 males showed a decrease in body condition with increasing number of parasites (estimated slope = −8.39; 95% CI = −14.6 to −2.2; *t* = −2.84, *P* = 0.005) while body condition of fish sired by exposed G1 males appeared relatively unaffected by parasite infection (estimated slope = 0.15; 95% CI = −5.1 to 5.4; *t* = −0.53, *P* = 0.59).

**Table 2:** Effects of paternal exposure and offspring infection intensity (number of established parasites) on individual body condition

Effect	d.f.	*F* value	*P*
Paternal exposure	1, 281	9.292	0.003
Paternal exp. × Offspring infection intensity	2, 281	4.116	0.017
Maternal half-sibship (random effect)	Variance = 36.88%		

The statistical table shows the outcome of a linear mixed model on individual body condition at the end of the experiment. The variation imputed to the random effect was estimated based on the ratio of the variance due to this effect over the total variance (d.f., degrees of freedom)

### Deciphering selection from increased parasite tolerance

The difference in tolerance of G2 fish with respect to paternal treatment indicates the existence of mechanisms induced by paternal infection. It could, however, also result from selection against low-quality G2 individuals during early life. Such a selection scenario could have resulted in an elevated average quality of the surviving G2 fish sired by exposed G1 males, and could, in turn, explain their elevated tolerance to parasite infection. To control for this scenario, we repeated the same statistical model as presented in Table 1, but we simulated selection by excluding G2 offspring sired by unexposed fathers across a range of selection strengths varying from 5 to 34.8% (the latter corresponding to twice the relative difference in overall survival between offspring from exposed and unexposed fathers, i.e. 17.4%). With these sensitive analyses, we simulated scenarios postulating: (1) selection against weaker G2 offspring sired by infected father (Table S7a) and (2) random selection independently of infection (Table S7b, S7c) to account for the effect that selection may not have exclusively removed the most susceptible individuals. In each case, based on 999 simulated subsets, we estimated the mean *P*-value and 95% CI of the paternal effect and the interaction between paternal exposure and offspring infection on offspring body condition. Paternal G1 treatment on offspring body condition remained significant, even at high levels of simulated random and infection-dependent selection (*P* < 0.047, Table S7). The interaction (G1 exposure × G2 infection) was supported by a statistical trend up to 20% contribution of selection (maximal mean *P*-value = 0.08, Table S7). Altogether, these analyses support the conclusion that selection in offspring of exposed fathers at the juvenile stage was not the only source for differences in offspring condition in our experiment, as well as corroborate a potential context dependence for the benefits of this paternal effect. Finally, we show that infection cost (i.e. mean difference in body condition between infected and uninfected individuals per family) did not significantly correlate with offspring mortality (see Table S3).

## Discussion

In addition to traditional genetic inheritance, parental effects are potent processes that can alter offspring phenotypes (Marshall & Uller 2007; Bonduriansky 2012; Burgess & Marshall 2014). Here, we present compelling experimental evidence for transgenerational effects of paternal parasite exposure on juvenile survival and offspring condition. While offspring of exposed sires generally suffered from reduced juvenile survival, suggesting parasite-mediated selection, the surviving offspring showed a significantly higher body condition than their counterparts from unexposed fathers. Interestingly, our in-depth analyses revealed that a fine-tuned interaction between selection and parental effects may result in a context-dependent advantage of this transgenerational effect where effects are strongest when both parental and offspring generations are exposed to similar selective pressures.

Firstly, not only had exposed males lower motile sperm concentration than unexposed males, but this also resulted in a lower rate of paternity in competitive situations. When both total sperm concentration and, as a result, the concentration of motile sperms were adjusted, differential fertilisation success was not observed anymore, demonstrating that this trait is condition dependent and represents a key functional link between infection and reproductive success during sperm competition. Secondly, in non-competitive *in vitro* experiments, male infection resulted in increased reproductive failures and lower probability for the offspring to reach adulthood. This result demonstrates that parasite exposure affects fertilisation and post-fertilisation development and although poor-quality sperm can fertilise eggs in a non-competitive interaction, carry-over effects can then exist. Altogether, we demonstrate strong sperm-mediated transgenerational costs of parasite infection on reproductive success. These results are consistent with: (1) studies showing that the activation of the immune system upon stimulation decreases sperm velocity and fertilisation success (Chargé *et al.* 2010; Losdat *et al.* 2011), (2) the *sick sperm* hypothesis, where paternal stress can alter sperm phenotype and affect post-zygotic development and performance (Crean *et al.* 2012, 2013; Rando 2012; Bromfield *et al.* 2014; Zajitschek 2014). Furthermore, zygote and juvenile mortality did not correlate among families. Whether this suggests several independent mechanisms or an interaction between genetic background and the expression of transgenerational costs on reproductive success remains an open question. Even though the direct mechanisms of sperm-mediated effects here are not clear, they may be associated with the release of reactive oxygen and nitrogen radicals which can damage proteins, lipids, DNA and can disrupt mitochondrial function (Sorci & Faivre 2009).

Using male siblings in our experimental design, we minimised the potential effects of classical genetics, e.g. through the inheritance of resistance alleles (Eizaguirre *et al.* 2012) and demonstrate that a large proportion of the observed costs originated from non-genetic paternal effects. To control for the possibility that treatment-induced selective mortality may have acted specifically against weaker offspring sired by exposed fathers and thus biased their mean intrinsic quality independent of paternal effects, we simulated varying levels of selection. While selection prior to parasite exposure of the offspring has probably acted in our experiment, our analyses also support the observation that increased body condition is associated with infection-induced paternal effects. Moreover, increased tolerance was not associated with high levels of mortality at the family level. Thus, both parasite resistance and tolerance are likely shaped by processes involving both genetic and non-genetic transgenerational effects.

Whether and when paternal effects are adaptive remain open questions in the literature (e.g. Uller *et al.* 2013). For paternal effects to be adaptive and thus get selected for, the benefits would have to outweigh the associated costs. In this study, we show that paternal infection can have significant costs ranging from deficient sperm to juvenile mortality, but paternal infection can also have clear beneficial effects on offspring condition, leading to a compensatory increase in Darwinian fitness of exposed fathers. The significant cost of infection in offspring sired by non-infected males is likely to lead to their competitive disadvantage against offspring sired by infected males, particularly as body condition is an accurate measurement of energy reserves and mate quality in sticklebacks (Milinski & Bakker 1990; Chellappa *et al.* 1995; Jakob *et al.* 1996). With our experimental design, we could also test the hypothesis that adaptive paternal effects are context dependent, i.e. expressed when both the paternal and offspring generations are predictably exposed to the same selective pressure. Burgess & Marshall (2014) recently stressed the importance of environmental predictability in the study of adaptive paternal effects. At least in our stickleback populations, the presence of a parasite in a given generation is more likely to predict parasite presence in the next generation than to predict parasite absence in the next generation (Kalbe *et al.* 2002; unpublished data). The fact that paternal effects are only observed in actually infected offspring may be due to the favourable laboratory conditions under which fish were kept and where costs associated to solely mounting an immune response (without the continuous costs of parasite infection) may be compensated for (Jäger *et al.* 2007). Nonetheless, our study suggests that under predictable selective pressures that impact both parental and offspring generations (such as parasite infection), transgenerational effects can be adaptive.

Interestingly, the paternal effects were not expressed as increased resistance to the parasite, but rather as a difference in body condition, resulting from increased tolerance (Råberg *et al.* 2007; Sorci 2013). In our experiments, offspring body condition had both a genetic and non-genetic transgenerational component, while the probability of infection and the level of infection strongly depended on the family background (i.e. classical genetics). Although our experimental design significantly reduced genetic variation, we still show that this variation played a major role in the individuals’ response to parasite infection.

There is substantial evidence for mechanisms of non-genetic inheritance, such as the inheritance of epigenetic alterations or the transmission of proteins or molecules from the parent to the offspring (Bonduriansky & Day 2009; Kappeler & Meaney 2010; Jiang *et al.* 2013). As males are more limited in their possibility to transmit information and resources than females (Curley *et al.* 2011), we expect transgenerational paternal effects to be mainly mediated through epigenetic changes in the germ line. Ultimately, epigenetic changes can also affect selection, e.g. by allowing for a more plastic and more immediate response to selection than classical genetic mechanisms of inheritance (Klironomos *et al.* 2013). Furthermore, parental effects may buffer selection at the genetic level. This can allow for the short-term maintenance of otherwise neutral or even slightly deleterious alleles, potentially promoting allelic diversity at genes involved in the response to fluctuating selective pressures, alongside traditional processes of long-term balancing selection. This can influence many processes such as population extinction, speciation and specifically host–parasite coevolutionary dynamics (Bernardo 1996; Wolf *et al.* 1998).
