# Supplementary material for: Experimental parasite infection reveals costs and benefits of paternal effects
Source: Ecol Lett. 2014 Aug 28;17(11):1409–17. doi: 10.1111/ele.12344 (PMC4282062; doi:10.1111/ele.12344)
Supplement: Supplementary file 1 — Supplementary [file ele0017-1409-SD1.docx]

Supporting Information for **Experimental parasite infection reveals context-dependent benefits of paternal effects** (Figure S1-S2 and Tables S1-S4)

Joshka Kaufmann^1^, Tobias L. Lenz^1, 2^, Manfred Milinski^1^, Christophe Eizaguirre^1, 3, 4^

^1^Max Planck Institute for Evolutionary Biology, Department of Evolutionary Ecology, Plön, 24306, Germany

^2^Division of Genetics, Brigham and Women’s Hospital, Harvard Medical School, Boston, MA 02115, USA

^3^GEOMAR, Helmholtz Centre for Ocean Research, Kiel, 24105, Germany

^4^Present address: School of Biological and Chemical Sciences, Queen Mary, University of London, London, E1 4NS, United Kingdom

**Supplementary figure S1**: Stickleback eggs

**Supplementary figure S2**: Trans-generational effects of paternal parasite exposure on infection probability

**Supplementary figure S3**: Trans-generational effects of paternal parasite exposure on body condition at the end of the experiment

**Supplementary table S1**: Differences in male mortality or reproductive behavior

**Supplementary table S2**: Test for overdispersion in Generalized Linear Mixed Models

**Supplementary table S3**: Correlation between life history traits

**Supplementary table S4**: Effects of paternal exposure on the likelihood of being infected

**Supplementary table S5**: Effects of paternal exposure on parasite load.

**Supplementary table S6**: Effects of paternal exposure, offspring exposure and sex on individual body condition.

**Supplementary table S7**: Effect of various selection strengths on statistical evidence for paternal effects.

**Supplementary figure S1**: Stickleback eggs at day 5 post fertilization: fully developed embryos, one dead undeveloped zygote and an unfertilized oocyte.


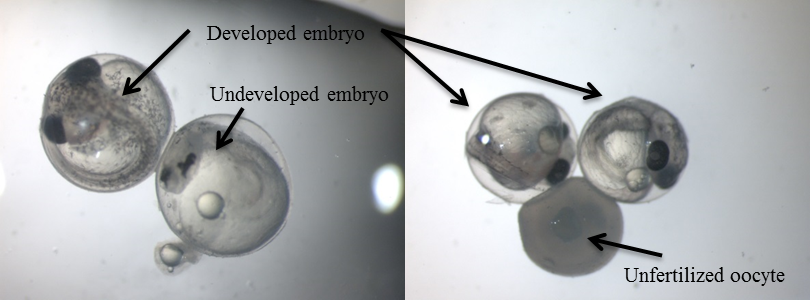


**Supplementary figure S2**: Trans-generational effects of paternal parasite exposure on infection probability in G2 fish generation (mean probability of being infected when exposed). Shown are means of infection probability (no significant differences) in G2 offspring sired by either exposed or unexposed fathers. Error bars represent ±1 SE.

**Supplementary figure S3**: Trans-generational effects of paternal parasite exposure on body condition at the end of the experiment. Bars are means (±1 SE) of body condition (residuals from the regression of body mass on body length) for offspring sired by either exposed or unexposed fathers. As offspring body condition is normalized around zero, the boxes represent the differences from the mean body condition in all offspring.

**Supplementary table S1**: Differences in male mortality or reproductive behavior.

Because parasite exposure is known to affect mortality and reproductive behaviour, our parasite exposure treatment in the G1 generation may be associated with unintended selection bias in male quality between the treatment groups. In order to test for this unintended bias, we tested for differences between exposed and unexposed G1 males in mortality, nest building behaviours, or the manifestation of courtship behaviour. Parasite exposure did not significantly affect the time needed to build a high quality nest (exposed males: 15.6 days ± SD 1.02, unexposed males: 15.2 days ± SD 1.10, t_1,121_=0.263, p=0.79). Given an effect size of d=1.372 (derived from Rushbrook & Barber (2006)), our sample size of 121 individuals ought to be sufficient to detect differences in nesting behaviour at α=0.05 with a probability of β=0.999 if it had existed. Furthermore, comparable experiments have reported significant effects on nest building behaviour with smaller sample sizes (Rushbrook & Barber 2006; Jäger *et al.* 2007; Macnab *et al.* 2009). Overall, 8 of the 71 exposed G1 males and 6 of the 62 unexposed G1 males were excluded or died over the course of the experiment (χ^2^=0.09, p=0.77), suggesting very limited (if any) bias in overall paternal quality resulting from the parasite treatment. Shown in table are the relative frequencies, the odds ratio for each category and the p-value associated with a Fisher exact test. Numbers in brackets represent the number of males that were not used in the experiment over the number of males affected in each treatment.

|  | **Exposed males** | **Unexposed males** | **Odds ratio** | **p-value** |
| --- | --- | --- | --- | --- |
| No nest building | 2.9 % (2/67) | 1.8 % (1/57) | 1.723 | 1 |
| No courtship behaviour | 3.1 % (2/65) | 0 % (0/56) | Inf. | 0.499 |
| Died | 5.6 % (4/71) | 8.1 % (5/62) | 0.681 | 0.733 |

**Supplementary table S2**: Test for over-dispersion in Generalized Linear Mixed Models

Because of the intrinsic distribution of parasites in a population, we tested for over-dispersion (excess of residual variance) in our two models including non-normal data (binomial and count) and mixed effects (maternal half-sibship effect). We did not find a significant difference between the models reported and the same models with an additional observation-level random effect (Infection probability: χ_1_=0, p=0.999, Infection intensity: χ _1_=0.001, p=0.975). In addition, we report in the following table the residual degrees of freedom (rdf), the sum of squared Pearson residuals (SS residuals), the ratio of SS residuals to rdf and the p-value based on the χ^2^ distribution. For each model, the ratio was close to 1 and no significant excess of variance was found.

|  | rdf | SS residuals | Ratio | p-value |
| --- | --- | --- | --- | --- |
| Infection rate model | 217 | 199.11 | 0.918 | 0.803 |
| Infection intensity model | 107 | 97.454 | 0.911 | 0.735 |

**Supplementary table S3**: Correlation between life history traits: cost of infection in exposed individuals, juvenile mortality, zygote mortality, motile spermatozoa [spz] concentration. Shown are Spearman Rho estimate (below the diagonal) and the associated p-value (above the diagonal). Motile sperm concentration was estimated at the father level and early life history traits were estimated at the family level (n=96-100 families). The cost of infection was calculated as the mean difference in body condition between infected and uninfected individuals per family (n=27 families).

|  | Infection cost | Juvenile mortality | Zygote mortality | Motile spz concentration |
| --- | --- | --- | --- | --- |
| Infection cost |  | 0.311 | 0.463 | 0.520 |
| Juvenile mortality | -0.24 |  | 0.084 | 0.128 |
| Zygote mortality | -0.15 | 0.17 |  | 0.511 |
| Motile spz concentration | -0.13 | 0.13 | 0.07 |  |

**Supplementary table S4**: Effects of paternal exposure on the likelihood of being infected.

Statistical table showing results of the likelihood ratio test between two generalized linear models with or without paternal infection as fixed factor. (d.f. : degrees of freedom, AIC : Akaike information criterion, BIC: Bayesian information criterion)

|  | d.f. | AIC | BIC | logLik | χ2 | P(>χ) |
| --- | --- | --- | --- | --- | --- | --- |
| Without paternal infection | 4 | 303.1 | 316.8 | -147.6 |  |  |
| With paternal infection | 6 | 303.5 | 323.9 | -145.8 | 3.599 | 0.165 |

| Variation due to maternal half sibship identity : 42.92% | |
| --- | --- |
| n=223 | groups=15 |

**Supplementary table S5**: Effects of paternal exposure on parasite load.

Statistical table showing results of the likelihood ratio test between two generalized linear models with or without paternal infection as fixed factor. (d.f. : degrees of freedom, AIC : Akaike information criterion, BIC: Bayesian information criterion)

|  | d.f. | AIC | BIC | logLik | χ2 | P(>χ) |
| --- | --- | --- | --- | --- | --- | --- |
| Without paternal infection | 4 | 110.3 | 121.21 | -51.15 |  |  |
| With paternal infection | 6 | 114.2 | 130.6 | -51.12 | 0.061 | 0.970 |

| Variation due to maternal half sibship identity : 19.07% | |
| --- | --- |
| n=113 | groups=15 |

**Supplementary table S6**: Effects of paternal exposure, offspring exposure (exposed vs. control) and sex on individual body condition. The table shows the outcome of a linear mixed effect model on individual body condition at the end of the experiment. The variation attributed to the random effect was estimated based on the ratio of the variance due to this effect over the total variance (d.f.: degrees of freedom).

| Effect | d.f. | F value | P |
| --- | --- | --- | --- |
| **Paternal exposure** | **1, 471** | **8.737** | **0.003** |
| **Offspring exposure** | **1, 471** | **6.418** | **0.012** |
| Offspring sex | 1, 471 | 0.267 | 0.606 |
| Paternal exposure x Offspring exposure | 1, 471 | 0.342 | 0.559 |
| Maternal half sibship (random effect) | 38.41% |  |  |

**Supplementary table S7**: Effect of various selection strengths on statistical evidence for paternal effects.

Linear mixed effect models on offspring body condition included the paternal exposure, offspring infection (exposed-infected vs. exposed-non-infected), sex and the interaction between paternal exposure and offspring infection. We simulated selection by excluding a percentage of G2 offspring sired by unexposed fathers to evaluate the strength of selection associated with early life mortality in offspring sired by exposed fathers. This percentage (i.e. selection levels) varied from 5 to 34.8 %, the latter representing twice the relative difference in overall survival between offspring from exposed and unexposed fathers (17.4%). Accordingly, each model was based on a subset of data after *in silico* mortality simulation.

In (a), we removed a proportion of the most infected offspring (based on infection rate) of unexposed fathers, conservatively postulating that selection could have removed the most susceptible offspring sired by exposed fathers. P-value estimates were based on the result of all possible subsets, as individuals with equal infection rates were also randomly excluded. In (b) and (c), we randomly excluded between 5 and 34.8% of either exposed (b) or infected (c) offspring sired by unexposed fathers. Mean p-values and 95% confidence intervals were calculated for each selection level and each scenario, based on 999 models for randomly produced subsets. We report p-values for the main paternal effect and for condition-dependence (interaction effect). Significant effects are highlighted in bold.

Results show that the main paternal effect was statistically robust, even at high levels of selection in every case scenario. The interaction effect was supported by statistical trends even at high levels of selection (up to 20%), showing a complex interplay between selection and paternal effects on the expression of parasite tolerance.

|  | (a) Selection based on high infection rate | | (b) Random selection amongst exposed | | (c) Random selection amongst infected | |
| --- | --- | --- | --- | --- | --- | --- |
| Selection level (%) | Main effect | Interaction | Main effect | Interaction | Main effect | Interaction |
| 0 | **0.005** | **0.043** | n/a | n/a | n/a | n/a |
| 5 | **0.007** | 0.055 | **0.007 ± 0** | 0.054 ± 0.001 | **0.006 ± 0** | **0.046 ± 0.001** |
| 10 | **0.010 ± 0.001** | 0.068 ± 0.011 | **0.009 ± 0** | 0.062 ± 0.002 | **0.007 ± 0** | **0.049 ± 0.001** |
| 15 | **0.008 ± 0.001** | 0.061 ± 0.005 | **0.012 ± 0.001** | 0.073 ± 0.003 | **0.009 ± 0** | 0.052 ± 0.001 |
| 17.4^*^ | **0.010 ± 0** | 0.071 ± 0.003 | **0.014 ± 0.001** | 0.080 ± 0.004 | **0.012 ± 0** | 0.059 ± 0.002 |
| 20 | **0.012 ± 0** | 0.081 ± 0.002 | **0.014 ± 0.001** | 0.082 ± 0.004 | **0.014 ± 0.001** | 0.063 ± 0.002 |
| 34.8^§^ | **0.047 ± 0.004** | 0.187 ± 0.014 | **0.030 ± 0.003** | 0.126 ± 0.008 | **0.023 ± 0.001** | 0.078 ± 0.003 |

*: Equivalent selection ; §: Double selection

References cited in Supplementary information:

1.

Jäger, I., Eizaguirre, C., Griffiths, S.W., Kalbe, M., Krobbach, C.K., Reusch, T.B.H., *et al.* (2007). Individual MHC class I and MHC class IIB diversities are associated with male and female reproductive traits in the three-spined stickleback. *J. Evol. Biol.*, 20, 2005–15.

2.

Macnab, V., Katsiadaki, I. & Barber, I. (2009). Reproductive potential of Schistocephalus solidus-infected male three-spined stickleback Gasterosteus aculeatus from two U.K. populations. *J. Fish Biol.*, 75, 2095–107.

3.

Rushbrook, B.J. & Barber, I. (2006). Nesting, courtship and kidney hypertrophy in Schistocephalus-infected male three-spined stickleback from an upland lake. *J. Fish Biol.*, 69, 870–882.
